# A comparison of efficacy and safety of complementary and alternative therapies for children with asthma: A protocol for systematic review and meta analysis

**DOI:** 10.1097/MD.0000000000024946

**Published:** 2021-03-19

**Authors:** Peng Zhou, Leiming Xi, Hongan He, Baoqing Zhang, Yanning Li

**Affiliations:** a The First College of Clinical Medicine, Shandong University of Traditional Chinese Medicine; b The Affiliated Hospital of Shandong University of Traditional Chinese Medicine.

**Keywords:** children with asthma, complementary and alternative therapy, net meta-analysis, protocol, systematic review

## Abstract

**Background:**

Bronchial Asthma is a chronic, hyperreactive inflammation of the airway that involves a variety of inflammatory cells. Due to the persistence of airway hyperresponsiveness, lung function is progressively damaged, making asthma more stubborn and difficult to heal. In recent years, the prevalence of childhood asthma is still on an increasing trend. Repeated asthma attacks not only affect children's life and learning, but also bring greater economic and mental burden to children's families, and even threaten children's lives. Traditional treatment methods such as oral western medicine, atomization therapy has obvious limitations, and the complementary and alternative therapy is an effective method to treat asthma in children. This study will evaluate the efficacy and safety of various complementary and alternative therapies for children with asthma by means of mesh meta-analysis. In order to provide the basis for clinical rational use.

**Methods:**

Use the computer to search the self-built database until January 2021, the China National Knowledge Infrastructure, Wanfang Database, Chinese Scientific Journals Database, China Biomedical Literature Database, PubMed, Cochrance Library, EMBASE, Web of Science, Clinical Trials and other electronic databases to collect RCT studies on complementary and alternative therapies for for children with asthma. We will screen the relevant literature included in the systematic review/meta analysis. At the same time, Revman 5.3 software will be used for meta-analysis, and grade will be used to grade the quality of evidence in the network meta-analysis.

**Results:**

This study will compare the efficacy and safety of different complementary and alternative therapies to treat childhood asthma, and evaluate and rank different interventions.

**Conclusion:**

The combined use of complementary and alternative therapies for childhood asthma on the basis of conventional basic treatment can improve clinical efficacy, reduce the occurrence of adverse reactions, improve the quality of life of children, and provide strong support for the rational use of clinicians.

**INPLASY registration number:**

INPLASY202120005

## 1 Introduction

Bronchial Asthma is a chronic, hyperreactive inflammation of the airway that involves a variety of inflammatory cells. The airway can be stimulated by a variety of factors, leading to spasm and contraction of the airway, showing recurrent cough, wheezing, shortness of breath, and expiratory dyspnea. The global prevalence of asthma and asthmatic diseases in children has been on the rise in recent years.^[1–3]^ Approximately 70% to 80% of bronchial asthma patients develop during childhood of 4 to 5 years.^[4,5]^ The clinical treatment of childhood asthma emphasizes on “anti-airway chronic inflammation.” The treatment methods include inhaled corticosteroids and/or bronchodilators, oral drugs and so on.^[6,7]^ Long term inhaled corticosteroids have a certain impact on the growth and development of children, there is immunosuppression and increase the risk of respiratory tract infection, and the hormone can not effectively inhibit the production and release of leukotrienes in inflammatory response. Some parents have poor compliance with long-term aerosol inhalation and oral medication, and the condition of many children's asthma is not ideal control.

Complementary and alternative therapy plays an important role in the adjuvant treatment of asthma in children, mainly including moxibustion, massage, Chinese herbal medicine, acupoint application, and so on.^[8–11]^ Complementary and alternative therapy for childhood asthma has been well practiced in clinic and plays an important role in delaying the onset of asthma and alleviating the disease.^[12,13]^ Studies have shown that acupuncture can significantly reduce the content of sIgA in the nose and saliva secretions of patients with allergic asthma, improve the lung function of patients with asthma, and expand the bronchus.^[14,15]^ Massage can enhance the excitability of the vagus nerve, reduce cortisol levels, relieve behavioral anxiety in young children, and improve lung function index.^[16,17]^ Chinese herbal medicine is widely used in the treatment of asthma in children, which has obvious effect in reducing the number of asthma attacks, reducing airway resistance and improving TCM syndrome score.^[18–20]^

At present, there are many complementary and alternative therapies for childhood asthma. Although randomized controlled trials and systematic reviews have evaluated and reported different treatment methods, there is still a lack of effective evidence-based medical evidence. Therefore, we will compare and evaluate various intervention measures to provide a powerful reference for clinicians to make reasonable choices. This paper will compare the efficacy and safety of a variety of complementary and alternative therapies for children with asthma through the method of network meta-analysis, so as to objectively evaluate the clinical efficacy of different complementary and alternative therapies, and look forward to exploring safer and more effective methods to relieve the symptoms of children with asthma.

## 2 Materials and methods

We will report in strict accordance with Preferred Reporting Items for Systematic Review and Meta Analysis Protocols.^[21]^

### 2.1 Study registration

The net meta-analysis has been registered on the International Platform of Registry System Review and Meta-analysis Protocols (INPLASY) and the registration number is INPLASY202120005 (URL = https://inplasy.com/inplasy-2021-2-0005/).

### 2.2 Inclusion criteria

#### 2.2.1 Research Type

Randomized controlled trials, whether to use blind method, and systematic review / meta-analysis of complementary and alternative therapies for childhood asthma, including moxibustion, massage, Chinese herbal medicine, acupoint application, etc.

#### 2.2.2 Participants

(1) Meet the relevant diagnostic criteria for bronchial asthma in children, and the
(2) severity of the illness is unlimited.
(3) The age is between 1 and 18 years old.
(4) There are no restrictions on gender and race.

#### 2.2.3 Intervention and comparison

In the treatment group, basic intervention measures were adopted, including moxibustion, massage, traditional Chinese medicine, acupoint application and other complementary and alternative therapies, on the basis of conventional treatment in western medicine. Different interventions can be used individually or in combination. The control group received conventional western medicine treatment.

#### 2.2.4 Outcomes

Outcome indicators:

(1) The effective rate standard is based on the efficacy index (that is, the difference between the scores before and after treatment/the percentage of the score before treatment). Healing is curative index>90%, markedly effective is curative index 61% to 90%, effective is curative index 30% to 60%, and invalid is curative index <30%. In addition, the outcome indicators also include disease recurrence rate, incidence of adverse reactions and so on;
(2) Pulmonary function indicators: forced expiratory volume at the end of the first second, forced vital capacity, forced expiratory volume at the end of the first second, percentage of forced vital capacity;
(3) The occurrence of adverse reactions.

### 2.3 Exclusion criteria

It is necessary to exclude:

(1) The trial design is not rigorous enough, and the control group is not strictly set;
(2) Literature with no outcome indicators available;
(3) Repeated published literature;
(4) Non clinical controlled trials.

### 2.4 Search strategy

The general search principle is based on subject terms and free words, and the search time limit is from the date of establishment of each database to January 2021. Computer search of electronic databases such as China Knowledge Network, Wanfang Database, Chinese Science Journal Database, China Biomedical Literature Database, PubMed, Cochrance Library, EMBASE, Web of Science, Clinical Trials, etc, to collect relevant information RCT study of complementary and alternative treatments for childhood asthma. Screen the relevant literature included in the systematic review/meta analysis, if there are differences of opinion during the selection and inclusion of the literature, we will resolve it through relevant discussions or through consultation with third-party researchers. And explain the reasons for the differences. The specific retrieval strategy of PubMed database is shown in Table 1.

**Table 1:** Detail of the search strategy for PubMed.

NO.	Search item
1#	“Asthma”[Mesh]
	(((Asthmas[Title/Abstract]) OR (Bronchial Asthma[Title/Abstract]) OR (Asthma, Bronchial[Title/Abstract])
2#	Asthma [Title/Abstract]))OR (Bronchial Asthma[Title/Abstract]))OR (Asthma, Bronchial[Title/Abstract])
3#	“Child”[Mesh]
4#	Children[Title/Abstract]
5#	1#OR 2# OR 3# OR 4#
6#	“Complementary Therapies”[Mesh]
7#	((((((((Therapies, Complementary[Title/Abstract]) OR (Therapy, Complementary[Title/Abstract])) OR (Complementary Medicine[Title/Abstract])) OR (Medicine, Complementary[Title/Abstract])) OR (Alternative Medicine[Title/Abstract])) OR (Medicine, Alternative[Title/Abstract])) OR (Alternative Therapies[Title/Abstract])) OR (Therapies, Alternative[Title/Abstract])) OR (Therapy, Alternative[Title/Abstract])
8#	6# OR 7#
9#	“Randomized Controlled Trials as Topic”[Mesh]
10#	((Clinical Trials, Randomized[Title/Abstract]) OR (Trials, Randomized Clinical [Title/Abstract])) OR (Controlled Clinical Trials, Randomized[Title/Abstract])
11#	9# OR 10#
12#	5#AND8#AND11#

### 2.5 Data extraction

After two researchers independently read the title and abstract, they first excluded the randomized controlled trials that obviously did not meet the inclusion criteria, and carefully read the full text of the studies that might meet the inclusion criteria. Methodological quality in the final included studies was evaluated item by item strictly according to the risk bias assessment catalog of the Cochrane Collaboration. All extraction processes and data will be recorded through Microsoft Excel 2019 software and relevant information will be extracted. The extracted content includes: literature title, author, time, random method, blinding, follow-up, effective and total number of cases, baseline level, diagnostic criteria, inclusion and exclusion criteria, intervention measures, efficacy evaluation criteria and indicators, safety Sex, long-term efficacy, and so on.

### 2.6 Risk of bias assessment

Evaluate the risk of bias in selected documents according to Cochrance Handanbook 5.1 evaluation criteria items and tools.^[22,23]^ Independent evaluation by 2 evaluators, cross-check after the completion of the evaluation, if there is a difference, it needs to be negotiated. The evaluation items are:

(1) Generation of random sequence;
(2) Allocation hiding;
(3) Implementation of blind method for researchers and subjects;
(4) Implementation of blind method for evaluation of outcome indicators;
(5) Complete result data Sex;
(6) Selective reporting of research results;
(7) Other sources of bias. The above items are evaluated as “high,” “low” and “unclear.”

### 2.7 Statistical analysis

The Rev Man 5.3 software provided by Cochrane Collaboration was used for statistical analysis. Binary variables were expressed by Odds Ratio, continuous variables were expressed by Mean Difference, and 95% confidence interval was used for interval estimation, with *P* <.05 as the difference was statistically significant. If *P* > .5 and *I*^2^ < 50% between the results of each study, then there is no statistical heterogeneity among the studies, and the results will be meta-analyzed using a fixed-effect model. If *P* ≤ .5 and *I*^2^ ≥50% between the research results, it indicates that there is heterogeneity, analyze the causes of heterogeneity, conduct subgroup analysis, and use fixed effect model for analysis. Also need to rule out obvious clinical heterogeneity. If the heterogeneity comes from a low-quality study, a sensitivity analysis of the results is performed.

### 2.8 Grading the quality of evidence

We will evaluate the quality of articles from the aspects of risk bias, directness, indirectness, inconsistency, imprecision, and publication bias.^[24]^

## 3 Discussion

Asthma is chronic inflammation of the airways caused by a combination of factors, which can easily lead to allergic sensitization, airway remodeling and airway hyperresponsiveness, which will gradually reduce the lung function and immunity of children, which will seriously affect the physical and mental health of children. Glucocorticoid is one of the commonly used drugs in the treatment of asthma, and montelukast sodium, as a selective leukotriene receptor antagonist, is a commonly used controlled drug, and is recommended for combined treatment of patients with poorly controlled inhaled corticosteroid therapy.^[22]^ In the actual treatment process, parents are worried about long-term aerosol inhalation of glucocorticoids and β2 receptor agonists and side effects after long-term oral drug treatment, which makes the compliance of children's asthma treatment poor.^[23]^

Studies have confirmed that supplementary replacement therapy has a positive effect on reducing the frequency of childhood asthma attacks, alleviating airway spasms, improving chest tightness, coughing and other symptoms, and shortening the course of the disease. However, the existing meta-analysis in clinical practice may only be limited to the comparison between two interventions, and there is still a lack of comparative evaluation between multiple interventions. The purpose of this study is to systematically compare the efficacy and safety of various complementary and alternative therapies for the treatment of childhood asthma through a network meta-analysis, and to further evaluate their respective efficacy and safety. Provide strong evidence for clinicians to choose reasonable treatment methods.^[25,26]^

## Author contributions

**Conceptualization:** Peng Zhou, Leiming Xi, Yanning Li.

**Data curation:** Peng Zhou, Leiming Xi, Hongan He, Baoqing Zhang.

**Methodology:** Peng Zhou, Leiming Xi, Baoqing Zhang.

**Project administration:** Peng Zhou, Baoqing Zhang, Yanning Li.

**Search strategy:** Leiming Xi, Hongan He, Baoqing Zhang.

**Statistical analysis:** Leiming Xi, Hongan He.

**Software:** Hongan He, Baoqing Zhang.

**Writing – original draft:** Peng Zhou.

**Writing – review & editing:** Yanning Li.
